# EZH2‐mediated repression of Dkk1 promotes hepatic stellate cell activation and hepatic fibrosis

**DOI:** 10.1111/jcmm.13153

**Published:** 2017-03-23

**Authors:** Yang Yang, Xiao‐xia Chen, Wan‐xia Li, Xiao‐qin Wu, Cheng Huang, Juan Xie, Yu‐xin Zhao, Xiao‐ming Meng, Jun Li

**Affiliations:** ^1^ The key of Laboratory Precision Medicine for Severe Autoimmune Diseases of Anhui Province Anhui Institute of Innovative Drugs Anhui Medical University Hefei China; ^2^ The Key Laboratory of Anti‐inflammatory and Immune Medicines Ministry of Education Hefei China; ^3^ Institute for Liver Diseases of Anhui Medical University Hefei China; ^4^ Department of Pharmacy Anhui No. 2 Province People's Hospital Hefei China

**Keywords:** Hepatic fibrosis, histone methylation, enhancer of zeste homologue 2, Dickkopf1, Wnt/β‐catenin pathway

## Abstract

EZH2, a histone H3 lysine‐27‐specific methyltransferase, is involved in diverse physiological and pathological processes including cell proliferation and differentiation. However, the role of EZH2 in liver fibrosis is largely unknown. In this study, it was identified that EZH2 promoted Wnt pathway‐stimulated fibroblasts *in vitro* and *in vivo* by repressing Dkk‐1, which is a Wnt pathway antagonist. The expression of EZH2 was increased in CCl_4_‐induced rat liver and primary HSCs as well as TGF‐β1‐treated HSC‐T6, whereas the expression of Dkk1 was reduced. Silencing of EZH2 prevented TGF‐β1‐induced proliferation of HSC‐T6 cells and the expression of α‐SMA. In addition, knockdown of Dkk1 promoted TGF‐β1‐induced activation of HSCs. Moreover, silencing of EZH2 could restore the repression of Dkk‐1 through trimethylation of H3K27me3 in TGF‐β1‐treated HSC‐T6 cells. Interestingly, inhibition of EZH2 had almost no effect on the activation of HSC when Dkk1 was silenced. Collectively, EZH2‐mediated repression of Dkk1 promotes the activation of Wnt/β‐catenin pathway, which is an essential event for HSC activation.

## Introduction

Hepatic fibrosis is a serious medical problem worldwide with high morbidity and mortality. Emerging evidence has indicated hepatic stellate cells (HSCs) play pivotal roles in hepatic fibrosis. In response to fibrotic injury, quiescent HSCs would lose the lipid content, and transform into myofibroblast‐like cells (MFB) which are highly proliferative, contractile and fibrogenic, thereby contributing to the progression of hepatic fibrosis. However, the underlying mechanisms controlling the proliferation and activation of HSCs are still obscure 1, 2. It has been demonstrated that exposure of HSCs to Transforming growth factor‐β1 (TGF‐β1) remarkably activates Wnt/β‐catenin pathway 3, 4. Knockdown of β‐catenin inhibited cell proliferation and α‐smooth muscle actin (α‐SMA) expression in activated HSCs 5. Moreover, overexpression of Dickkopf family (Dkk) especially Dickkopf‐1 (Dkk‐1), an antagonist of Wnt/β‐catenin signalling, is capable of suppressing primary cultured HSCs 6. These observations indicate that activation of Wnt/β‐catenin pathway significantly contributes to the development of hepatic fibrosis 7, 8.

Enhancer of zeste homologue 2 (EZH2), a methyltransferase, is associated with transcriptional suppression of target genes [9]. Hussain *et al*. demonstrated that EZH2 suppressed Dkk1 transcription through trimethylation of Histone H3 lysine 27 trimethylation (H3K27me3) in lung cancer 10. It has been reported that EZH2 is essential for the initiation and development of various cancers. Results from Mann's group revealed that the mRNA and protein levels of EZH2 were up‐regulated in activated HSCs compared with quiescent HSC. Importantly, administration with 3‐deazaneplanocin A (DZNep), an inhibitor of EZH2, could prevent the transdifferentiation of HSCs and the induction of Collagen I 11. However, the mechanism of EZH2‐mediated hepatic fibrosis remains to be determined. In this study, we hypothesize that EZH2‐mediated suppression of Dkk1 may promote hepatic fibrosis by activating Wnt/β‐catenin pathway.

## Materials and methods

### CCl_4_‐treated rat hepatic fibrosis model

Normal Sprague‐Dawley rats (200 ± 20 g) provided by the Experimental Animal Center of Anhui Medical University were used to establish the hepatic fibrosis model. All of the experimental procedures were reviewed and approved by University Animal Care and Use Committee. The rat hepatic fibrosis was generated in a 12‐week treatment with carbon tetrachloride(1:1) (CCl_4_; Shantou Xilong Chemistry Plant, Guangdong, China) which was diluted with olive oil (1 ml/kg) and was injected by intraperitoneal injection two times a week 12. Control rats were injected intraperitoneally with 1 ml/kg of olive oil per bodyweight at the same time intervals. Rats were killed 24 hrs after the last injection of CCl_4_ and liver tissues were harvested for the further study. The liver tissues were used for haematoxylin and eosin (H&E) and Masson staining after fixed with 10% formalin.

### Hepatic stellate cells isolation

Primary stellate cells were isolated from the livers of SD rats as previously described 13. Briefly, the liver was perfused in situ with PB, a kind of buffer solution containing NaCl, KCl, HEPES and NaOH, to purge the liver of blood. After digestion of the liver with collagenase IV (Sigma‐Aldrich, St. Louis, MO, USA) and Pronase (Sigma‐Aldrich), the liver was disrupted in 1% BSA solution. The dispersed cell suspensions were layered by Nycodenz (Sigma‐Aldrich) density gradient centrifugation according to manufacture protocol. The resulting upper layer contained the freshly isolated HSCs.

### Cell culture

HSC‐T6 cells (Keygen Biological Technology, Nanjing, China) were cultured on plastic in Dulbecco's modified Eagle's medium (DMEM), supplemented with 100 U/ml penicillin and streptomycin and 10% foetal calf serum (FBS; Emd Millipore, Billerica, MA, USA). Cells were maintained at an atmosphere of 5% CO_2_ and 37°C. HSC‐T6 cells were cultured for 48 hrs and serum‐starved with 0.5% FBS for 24 hrs before adding TGF‐β1 10 ng/ml (Peprotech, Rocky Hill, NJ, USA).

### Cell proliferation assay

Cell proliferation assay was measured by using MTT assay (Sigma‐Aldrich). The cells were seeded into each well of 96‐well plates at a density of 5 × 10³ cells per well and cultured with various concentrations of DZNep (Selleckchem, Houston, TX, USA) and TGF‐β1 for 12, 24, 48 and 72 hrs. After culture, 20 μl (5 mg/ml) MTT reagent diluted by phosphate‐buffered saline (PBS) was added and incubated at 37°C for another 4 hrs. Then, the cells were dissolved in 150ul of dimethyl sulfoxide (DMSO; Sigma‐Aldrich). The optical density (OD) was determined at a single wavelength of 492 nm with Thermomax microplate reader (Bio‐Tek EL, Vermont, USA).

### Immunohistochemistry

Liver tissues were fixed in 4% paraformaldehyde, embedded in paraffin and stained for routine histology. The sections were deparaffinized in xylene and dehydrated in alcohol, and antigen retrieval was achieved for 15 min. by microwaving in citric saline. Endogenous peroxidase activity was blocked by 0.3% hydrogen peroxide pretreatment for 15 min. and then further blocked by using 5% bovine serum albumin. Then, the sections were incubated overnight at 4°C with rabbit monoclonal antibody against EZH2 (Cell Signaling, Boston, MA, USA, 1:50) and rabbit monoclonal antibody against α‐SMA (1:200). After rinsing by PBS, the sections were incubated at room temperature with biotinylated secondary antibody (Cowin Bioscience, Beijing, China) for 30 min. EZH2 and α‐SMA expressions were visualized by 3, 3′‐diaminobenzidine tetrahydrochloride (DAB; Cowin Bioscience) staining. Slides were counterstained with haematoxylin before dehydration and mounting, and EZH2 and α‐SMA‐positive areas within the fibrotic region were then observed.

### Immunocytochemistry

Cultured HSC‐T6 cells were treated with or without TGF‐β1 for 48 hrs and fixed with acetone. Staining was performed with rabbit anti‐Dkk1 (Abcam, Cambridge, UK). Counterstaining of nuclei was performed with 4′, 6‐diamidino‐2‐phenylindole (DAPI, Beyotime, Shanghai, China). Fluorescence intensity was analysed using IMAGE J (National Institutes of Health, MD, U.S.A) software (V.1.42).

### Quantitative real‐time PCR

Total RNA was extracted from rat livers and from HSC‐T6 cells using TRIzol reagent (Invitrogen, Carlsbad, CA, USA). The first‐strand cDNA was synthesized from total RNA by using Thermoscript RT‐PCR System (TaKara,Tokyo, Japan) according to the manufacturer's protocol. Relative levels of mRNA were determined with a SYBR Green using quantitative real‐time PCR detection system (Themal 5100). Quantitative real‐time PCR analyses of EZH2, Dkk1, β‐catenin and α‐SMA were performed, and the mRNA levels of β‐actin were used as an endogenous control for normalization. Real‐time PCR was carried out under a standard protocol using the following primers: EZH2 (forward: 5′‐GCACAGCAGAAGAACTGAAAGAAAA‐3′; reverse: 5′‐TCGACAAAAGAGCGTATGAAATGAA‐3′), Dkk1(forward:5′‐TGCTACATTGTCTCGTTTCTCTTG‐3′; reverse: 5′‐TTAGATGCCTATCATTGACTATTCC‐3′), β‐catenin (forward: 5′‐CTTA CGGCAATCAGGAAAGC‐3′; reverse: 5′‐ACAGACAGCACCTTCAGCACT‐3′), α‐SMA (forward: 5′‐CGAAGCGCAGAGCAAGAGA‐3′; reverse: 5′‐CATGTCGTC CCAGTTGGTGAT‐3′), β‐actin (forward: 5′‐CCCATCTATGAGGGTTACGC‐3′; reverse: 5′‐TTTAATGTCACGCACGATTTC‐3′). Real‐time PCR conditions were as follows: 95°C for 5 min., then 40 cycles of 95°C for 15 sec., 60°C for 30 sec., 72°C for 30 sec., then a final extension at 72°C for 30 sec. The fold change for mRNA relative to β‐actin was determined by the formula: 2^−ΔΔCt^. The relative mRNA expression was performed from three different experiments.

### RNA interference analysis

HSC‐T6 cells were transfected with 100 nM of small interfering RNA (siRNA) using Lipofectamine 2000 (Invitrogen) according to the manufacturer's instructions. The oligonucleotide sequences were as follows: EZH2‐siRNA (sense: 5′‐GGGCAUCUUUAUCAAAGAUTT‐3′ and antisense: 5′‐AUCUUUGAUAAAGA UGCCCTT‐3′); Dkk1‐siRNA (sense: 5′‐GCGUGGGAGGUGUACAAAUTT‐3′ and antisense: 5′‐AUUUGUACACCUCCCACGCTT‐3′). A negative scrambled siRNA (GenePharma, Shanghai, China) was used in parallel. Cells were cultured at 37°C for 6 hrs, and then, Q‐PCR and Western blotting were used 48 hrs after siRNA transfection.

### Western blot analysis

Rat liver tissues and HSC‐T6 cells were lysed with RIPA lysis buffer (Beyotime). Whole‐cell extracts were prepared, and protein concentration of samples was detected by using a BCA protein assay kit (Boster Bio, Wuhan, China). Whole‐cell extracts (30 or 50 mg) were then fractionated by electrophoresis through 10% or 12% sodium dodecyl sulphate‐polyacrylamide gel electrophoresis (SDS‐PAGE). Gels were run at 80 V for 30 min. followed by 120 V for 90 min. before being transferred onto a PVDF membrane (Millipore Corp). The membranes were then incubated in TBST containing 5% skim milk at 37°C for 3 hrs, and with specific primary antibodies at 4°C overnight. Rabbit monoclonal antibody recognizing EZH2 (Cell Signaling) was used 1:500, rabbit monoclonal anti‐Dkk1 (Abcam) was diluted 1:400, rabbit monoclonal anti‐β‐catenin, C‐myc, CyclinD1, H3K27me3 and H3 (Cell Signaling) were diluted 1:500, and rabbit monoclonal anti‐β‐actin and α‐SMA were diluted 1:500. Following incubation with the primary antibody, membranes were washed three times in TBS/Tween‐20 before incubation for 1 hr with goat anti‐rabbit horseradish peroxidase conjugated antibody at a 1:9000 dilution in TBS/Tween‐20 containing 5% milk. After washing in TBS/Tween‐20, the blots were processed with distilled water for detection of antigen using the enhanced chemiluminescence system. Proteins were visualized with the ECL chemiluminescent kit (ECL‐plus; Thermo Scientific, Waltham, MA, USA).

### Statistical analysis

Data are represented as Mean ± S.E. The analysis of statistics was performed by using analysis of variance (anova) tests and Student's *t*‐test for comparison between means, and one‐way analysis of variance with a *post hoc* Dunnett's test. Significance was defined as *P, ^#^ *P*, ^&^ *P* < 0.05, **P, ^##^ *P* < 0.01.

## Results

### Expressions of EZH2 and Dkk1 in the liver and HSC isolated from rats with CCl_4_‐treated hepatic fibrosis

**Figure 1:**
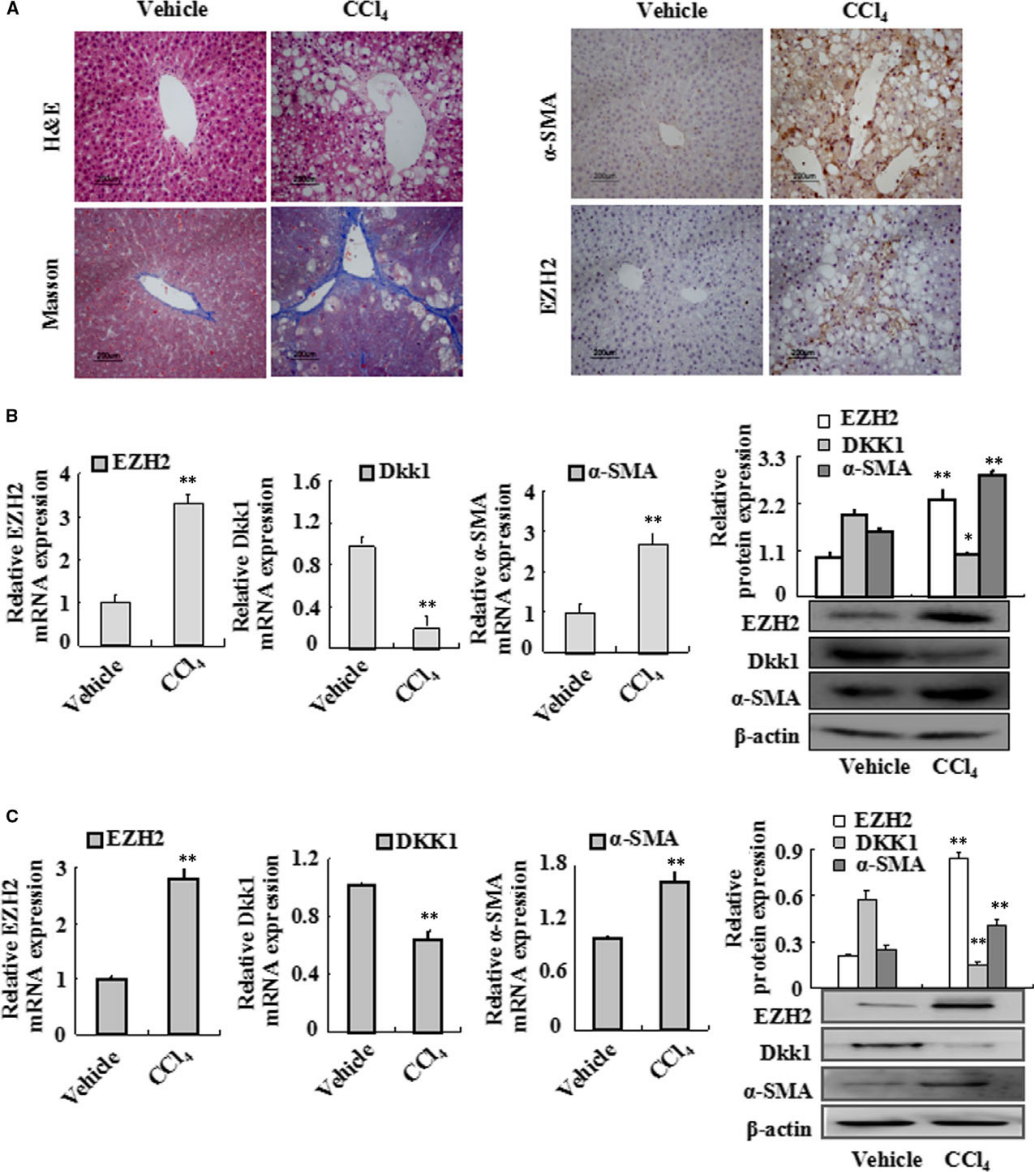
EZH2, α‐SMA and Dkk1 expressions in fibrotic rat liver tissues and primary HSC. (**A**) Vehicle liver tissues and fibrotic liver tissues were detected by haematoxylin and eosin (H&E) staining (×50) and Masson staining (×50). EZH2 and α‐SMA immunostaining of liver sections from vehicle‐treated rats and CCl4‐treated rats. Representative views from each group are presented (original magnification, ×50). (**B**) Total RNAs were isolated from the liver tissues of vehicle‐treated rats and CCl4‐treated rats, and real‐time PCR was performed to examine the mRNA levels of EZH2, Dkk1 and α‐SMA. Whole‐cell extracts were isolated from vehicle and fibrotic liver tissues, and Western blotting was performed to examine the protein levels of EZH2, Dkk1 and α‐SMA. (**C**) Total RNAs were isolated from primary HSC of vehicle‐treated rats and CCl4‐treated rats, and real‐time PCR was performed to examine the mRNA levels of EZH2, Dkk1 and α‐SMA. Whole‐cell extracts were isolated from primary HSC of vehicle‐treated rats and CCl4‐treated rats, and Western blotting was performed to examine the protein levels of EZH2, Dkk1 and α‐SMA. Data are representative of three independent experiments. *P < 0.05, **P < 0.01 *versus* vehicle.

To investigate the effect of histone methylation modification on hepatic fibrosis in CCl4‐treated rats, experiment was performed. Immunohistochemical results indicated that the expression of EZH2 was dramatically increased in the liver of CCl_4_‐treated rats (Fig. 1A). Quantitative real‐time PCR and Western blots analysis also demonstrated that the expression of EZH2 in CCl_4_‐treated group was higher than that in the vehicle group, while Dkk1 was down‐regulated (Fig. 1B). Similar results were found in HSCs isolated from the liver in model group (Fig. 1C). These results suggest that both EZH2 and Dkk1 might play an essential role in the pathogenesis of hepatic fibrosis in rat.

### Expressions of EZH2 and Dkk1 in TGF‐β1‐treated HSC‐T6 in vitro

**Figure 2:**
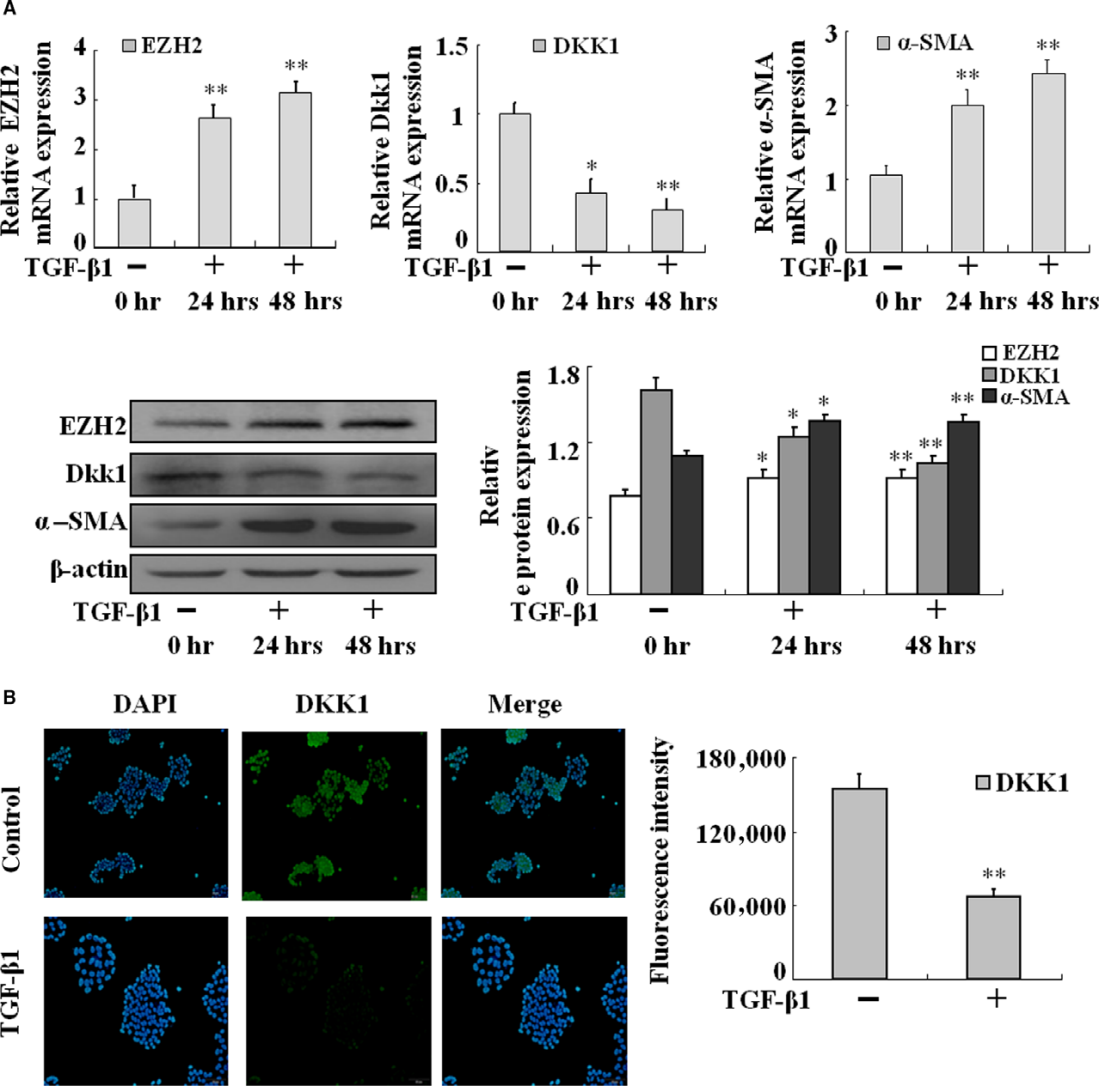
Up‐regulation of EZH2 and down‐regulation of Dkk1 in HSC‐T6 cells with the treatment of TGF‐β1. (**A**) HSC‐T6 cells induced by TGF‐β1 at three time points then total RNAs were isolated and real‐time PCR was performed to examine the mRNA levels of EZH2, Dkk1 and α‐SMA. HSC‐T6 cells induced by TGF‐β1 at three time points then whole‐cell extracts were isolated and Western blotting was performed to examine the protein levels of EZH2, Dkk1 and α‐SMA. Data are representative of three independent experiments. (**B**) HSC‐T6 cells treated with TGF‐β1 for 48 hrs, and then fixed in acetone, were used to the fluorescence staining of Dkk1. Data are representative of three independent experiments. *P < 0.05, **P < 0.01 *versus* 0 hr.

Next, we examined the expression profiles of EZH2 and Dkk1 in TGF‐β1‐treated HSCs *in vitro*. The mRNA and protein levels of EZH2 and α‐SMA were up‐regulated in TGF‐β1‐activated HSC‐T6 cells, whereas Dkk1 was down‐regulated (Fig. 2A). These findings were further confirmed by immunocytochemistry (Fig. 2B). Therefore, *in vitro* evidences further provide that EZH2 and Dkk1 may be involved in HSC activation *in vitro*.

### Inhibition of EZH2 prevents TGF‐β1‐mediated activation of HSC‐T6 cells

**Figure 3:**
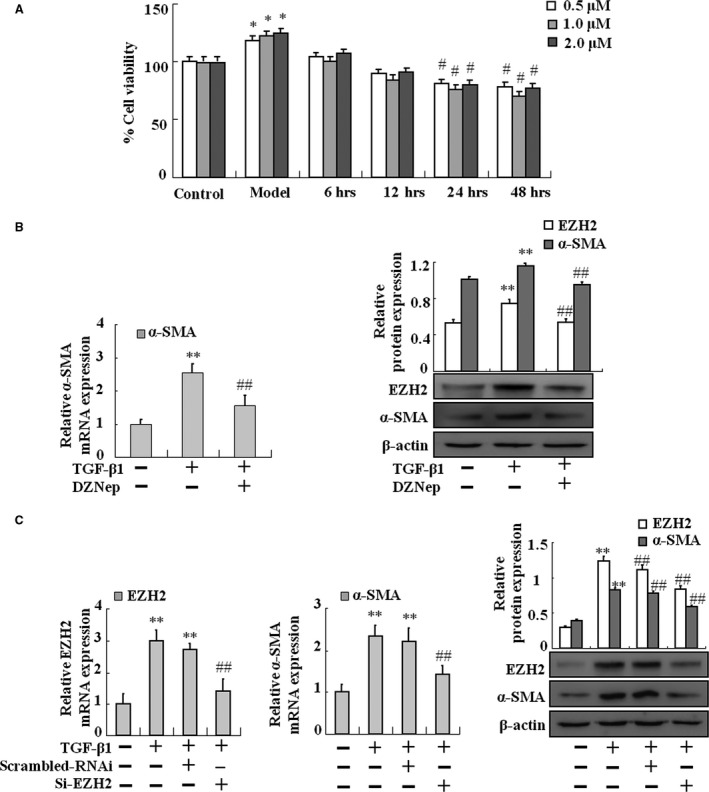
Effects of DZNep and EZH2 silencing on TGF‐β1‐treated HSC‐T6 activation. (**A**) MTT assay was performed to assess the effects of DZNep on TGF‐β1‐treated HSC‐T6 proliferation in different dosages and time points. (**B**) HSC‐T6 cells were treated with TGF‐β1 or TGF‐β1 plus DZNep. Real‐time PCR was performed to assess the mRNA expression level of α‐SMA at the three groups. HSC‐T6 cells were treated with TGF‐β1 or TGF‐β1 plus DZNep. Western blotting was carried out to assess the protein levels of EZH2 and α‐SMA at the three groups. (**C**) HSC‐T6 cells treated by TGF‐β1 with or without siRNA. Real‐time PCR was carried out to assess the mRNA expression levels of EZH2 and α‐SMA at the four groups. HSC‐T6 cells treated by TGF‐β1 with or without siRNA. Western blotting was carried out to assess the protein levels of EZH2 and α‐SMA at the four groups. Data are representative of at least three independent experiments. *P < 0.05, **P < 0.01 *versus* control, #*P* < 0.05, ##*P* < 0.01 *versus* model or scrambled RNAi.

We first examined the function of EZH2 in TGF‐β1‐treated HSC‐T6 cells with or without its inhibitor DZNep, respectively. As shown in Figure 3A, treatment with DZNep had a substantial inhibitory effect on cell proliferation in TGF‐β1‐stimulated HSCs. Furthermore, real‐time PCR and Western blot analysis revealed that DZNep could repress the expression of α‐SMA (Fig. 3B).

To further confirm the key role of EZH2 in TGF‐β1‐mediated HSC activation, knockdown of EZH2 by siRNA was performed. As shown in Figure 3C, the expression of EZH2 and α‐SMA was markedly up‐regulated in TGF‐β1‐treated HSC‐T6 cells. Nevertheless, TGF‐β1 failed to promote the expression of EZH2 and α‐SMA in EZH2‐silenced HSCs (Fig. 3C). Taken together, TGF‐β1‐mediated activation of HSC‐T6 might be in an EZH2‐dependent manner.

### Knockdown of Dkk1 promotes TGF‐β1‐mediated HSC‐T6 activation

**Figure 4:**
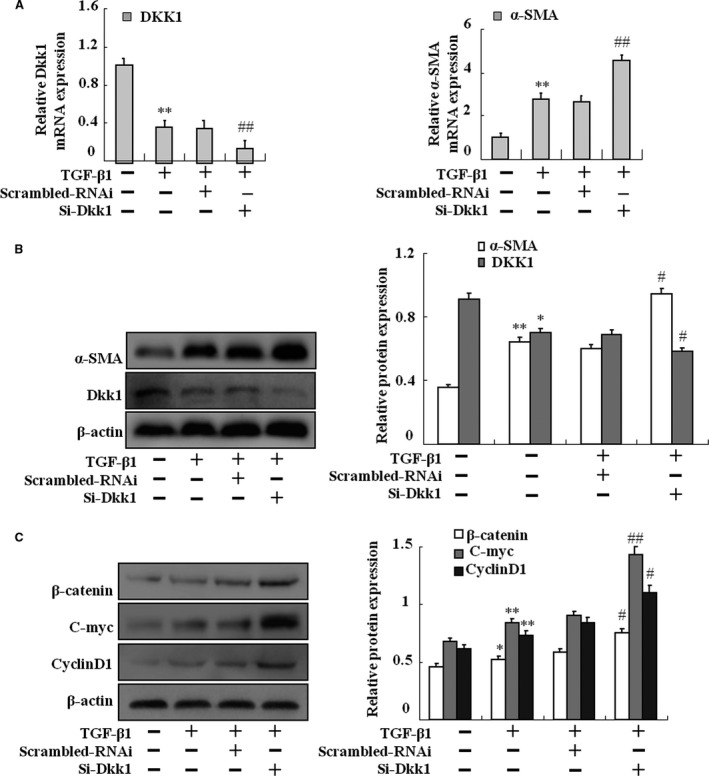
Effects of Dkk1 silencing on TGF‐β1‐treated HSC‐T6 activation. After transfection with Dkk1 siRNA or scrambled RNAi, HSC‐T6 cells were exposed to TGF‐β1 for 2 days. (**A**) HSC‐T6 cells treated by TGF‐β1 with or without siRNA then total RNAs were isolated and real‐time PCR was performed to examine the mRNA levels of Dkk1 and α‐SMA. (**B**) HSC‐T6 cells treated by TGF‐β1 with or without siRNA then whole‐cell extracts were isolated and Western blotting was performed to examine the protein levels of Dkk1, β‐catenin, C‐myc and CyclinD1. (**C**) HSC‐T6 cells treated by TGF‐β1 with or without siRNA then whole‐cell extracts were isolated and Western blotting was performed to examine the protein levels of α‐SMA. Data are representative of at least three independent experiments. *P < 0.05, **P < 0.01 *versus* control; #*P* < 0.05, ##*P* < 0.01 *versus* model or scrambled RNAi.

We also investigated whether Dkk1 had an effect on HSC‐T6 activation. Dkk1‐specific siRNA was applied in HSC‐T6 stimulated with TGF‐β1. As shown in Figure 4A and B, knockdown of Dkk1 in activated HSC‐T6 significantly attenuated the mRNA and protein expression of α‐SMA. Similar results were found in the expression of the critical protein β‐catenin in Wnt/β‐catenin pathway (Fig. 4C). These results suggest that Dkk1 might function as a suppressor in TGF‐β1‐induced activation of HSCs.

### Epigenetic modification is involved in the transcription of Dkk1

**Figure 5:**
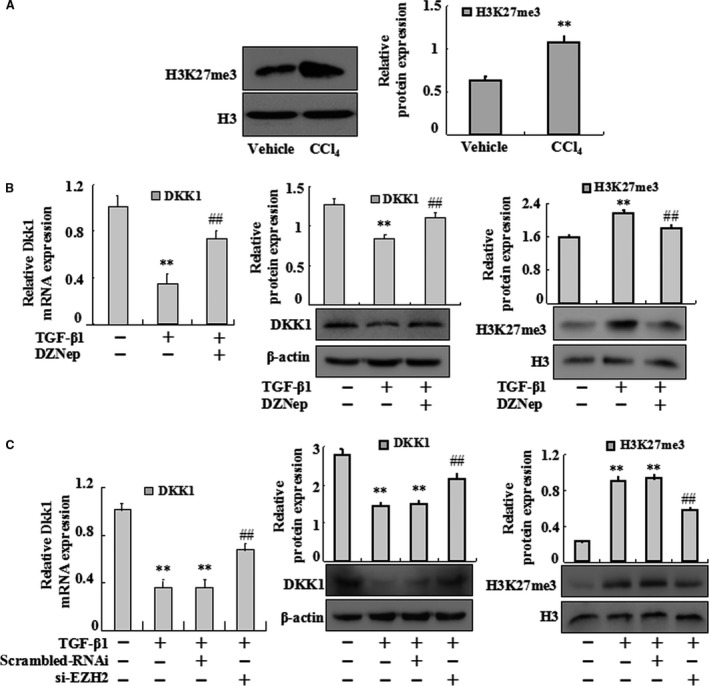
EZH2 inhibition has effects on Dkk1 expression in TGF‐β1‐treated HSC‐T6 activation. (A) Whole‐cell extracts were isolated from primary HSC and Western blotting was performed to test the protein levels of H3K27me3. Data are representative of at least three separate experiments. Data are representative of at least three independent experiments. **P < 0.01 *versus* vehicle. (**B**) HSC‐T6 cells were treated with TGF‐β1 or TGF‐β1 plus DZNep. Real‐time PCR was carried out to assess the mRNA expression levels of Dkk1. HSC‐T6 cells were treated with TGF‐β1 or TGF‐β1 plus DZNep. Western blotting was carried out to assess the protein levels of Dkk1. HSC‐T6 cells were treated with TGF‐β1 or TGF‐β1 plus DZNep. The protein levels of H3K27me3 were performed. (**C**) HSC‐T6 cells treated by TGF‐β1 with or without siRNA then total RNAs were isolated and real‐time PCR was performed to examine the mRNA levels of Dkk1. HSC‐T6 cells treated by TGF‐β1 with or without siRNA then whole‐cell extracts were isolated and Western blotting was performed to examine the protein levels of Dkk1. HSC‐T6 cells treated by siRNA with or without TGF‐β1 then whole‐cell extracts were isolated. The protein levels of H3K27me3 were performed. Data are representative of three independent experiments. **P < 0.01 *versus* control; ##*P* < 0.01 *versus* model or scrambled RNAi.

To further examine the mechanisms underlying the down‐regulated expression of Dkk1, the state of histone methylation was analysed *in vivo* and *in vitro*. We first examined the protein levels of H3K27me3 in primary HSCs in a rat model of CCl_4_‐treated hepatic fibrosis. As shown in Figure 5A, Western blot analysis revealed that H3K27me3 was simultaneously increased in model group. To confirm these findings seen in primary HSCs, HSC‐T6 cells were induced with TGF‐β1 and then treated with 1 μM DZNep for 24 hrs. Results showed that treatment with DZNep decreased the expression of H3K27me3 but increased the mRNA and protein expression of Dkk1 (Fig. 5B). Similar results were further confirmed by siRNA‐EZH2 in HSC‐T6 cells (Fig. 5C). Collectively, these results suggest that a decrease in Dkk1 expression was correlated to epigenetic mechanisms such as histone methylation in HSC activation.

### Essential role of Dkk1 in EZH2‐mediated activation of HSCs

**Figure 6:**
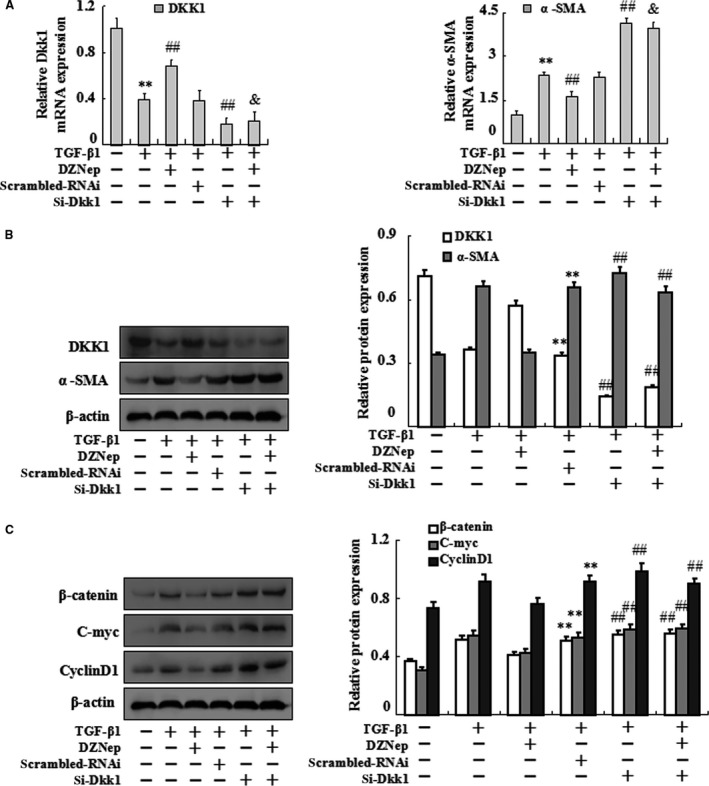
Effects of introducing Dkk1 siRNA and DZNep simultaneously on TGF‐β1‐treated HSC‐T6 activation. HSC‐T6 cells were divided into six groups, untreated HSC‐T6 cells (control groups), TGF‐β1‐treated HSC‐T6 cells (model groups), DZNep plus TGF‐β1‐treated HSC‐T6 cells, siControl transferation plus TGF‐β1‐treated HSC‐T6 cells, Dkk1 siRNA transferation plus TGF‐β1‐treated HSC‐T6 cells and Dkk1 siRNA transferation plus DZNep and TGF‐β1‐treated HSC‐T6 cells. (**A**)Real‐time PCR was carried out to assess the mRNA expression levels of Dkk1 and α‐SMA at the six groups. (**B**) Western blotting was carried out to assess the protein levels of Dkk1, α‐SMA at the six groups. (**C**) Western blotting was carried out to assess the protein levels of β‐catenin, C‐myc and CyclinD1 at the six groups. Data are representative of three independent experiments. **P < 0.01 *versus* control; ##*P* < 0.01 *versus* scrambled RNAi.

To confirm the essential role of Dkk1 in TGF‐β1‐treated HSC‐T6 cells, we silenced Dkk1 with or without DZNep. Real‐time PCR and Western blot analysis showed that there were no significant differences in the mRNA and protein expression levels of Dkk1 and α‐SMA between Dkk1‐siRNA and siRNA+DZNep‐treated group (Fig. 6A and B). In addition, the protein expression levels of β‐catenin, c‐myc and cyclinD1 also had no differences between these two groups (Fig. 6C).

### Epigenetic repression of Dkk1 contributes to the activation of Wnt/β‐catenin pathway

**Figure 7:**
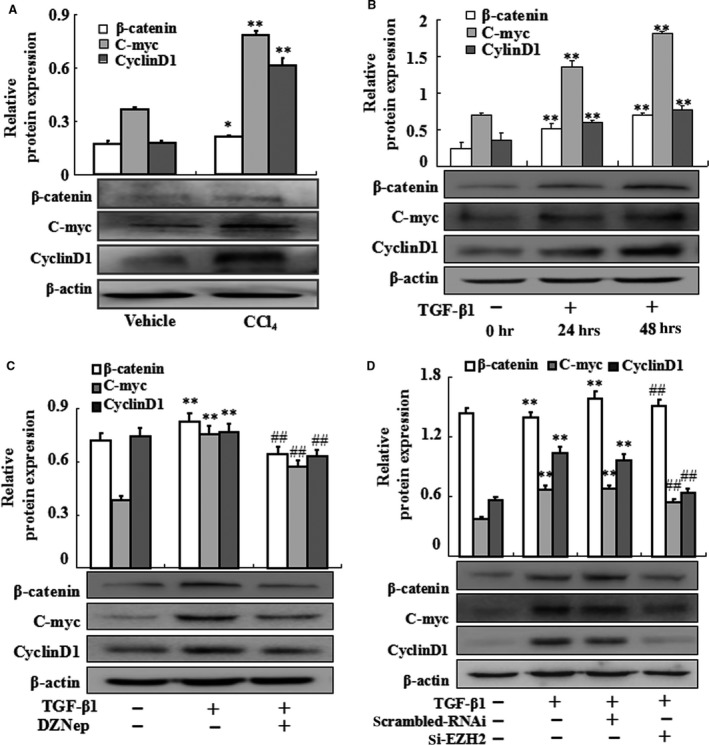
Effects of epigenetic silencing of Dkk1 on the expression of β‐catenin, C‐myc and CyclinD1. (**A**) The whole‐cell primary HSC extracts were isolated and Western blotting was performed to test the protein levels of β‐catenin, C‐myc and CyclinD1. Data are representative of at least three independent experiments. *P < 0.05, **P < 0.01 *versus* vehicle. (**B**) HSC‐T6 cells induced by TGF‐β1 at three time points then whole‐cell extracts were isolated and Western blotting was performed to examine the protein levels of β‐catenin, C‐myc and CyclinD1. (**C**) HSC‐T6 cells were treated with TGF‐β1 or TGF‐β1 plus DZNep. The protein levels of β‐catenin, C‐myc and CyclinD1 were performed. (**D**) HSC‐T6 cells treated by siRNA with or without TGF‐β1 then whole‐cell extracts were isolated and Western blotting was performed to examine the protein levels of β‐catenin, C‐myc and CyclinD1. Data are representative of at least three independent experiments. **P < 0.01 *versus* control; ##*P* < 0.01 *versus* model or scrambled RNAi.

To explore the effect of EZH2 on Wnt/β‐catenin signalling pathway, the expression of β‐catenin, c‐myc and cyclinD1 was analysed *in vivo* and *in vitro*. We first examined the protein levels in primary HSCs in a rat model of CCl_4_‐treated hepatic fibrosis. As shown in Figure 7A, Western blot analysis revealed that β‐catenin, C‐myc and CyclinD1 were simultaneously up‐regulated in model group. Similar results were observed in TGF‐β1‐treated HSC‐T6 (Fig. 7B). Furthermore, Western blot analysis revealed that the expression of β‐catenin, C‐myc and CyclinD1 was remarkably decreased in activated HSC‐T6 cells treated with DZNep (Fig. 7C). Similarly, siRNA EZH2 dramatically inhibited the expression of β‐catenin, C‐myc and CyclinD1 (Fig. 7D).

## Discussion

Activation and transdifferentiation of HSCs into myofibroblasts, characterized by accelerated cell proliferation and over‐production of Extracellular matrix (ECM), are the central event in hepatic fibrogenesis. Emerging evidence indicates that blockage of HSC activation/proliferation is an important treatment strategy for hepatic fibrosis 14, 15. However, the molecular mechanisms that regulate HSC activation and proliferation remain largely unclear. In this study, we firstly identified EZH2‐activated Wnt/β‐catenin pathway by repressing Dkk1, thereby acts as a key regulator of HSC activation *in vitro* and *in vivo*.

It is known that Wnt/β‐catenin signalling pathway is associated with the proliferation of various cancers, including hepatocellular carcinoma, suggesting that Wnt/β‐catenin signalling plays an important role in cell proliferation 16. Similarly, Wnt/β‐catenin signalling pathway plays a crucial role in the progression of fibrosis 17, 18. Accumulating evidence suggested that Wnt signalling was activated in HSCs during the progression of hepatic fibrosis, and some downstream elements of Wnt signalling pathway were induced in this process 19, 20. The important role of β‐catenin, a key regulator in the canonical Wnt/β‐catenin signalling pathway, has been clarified in liver development, remodelling and HSC activation 5, which was supported by our findings that the expression of β‐catenin was increased in primary HSCs isolated from the liver of CCl_4_‐treated rats and TGF‐β1‐treated HSCs *in vivo* and *in vitro*. Dkk1, an antagonist of Wnt/β‐catenin signalling, is capable of suppressing the expression of β‐catenin and the activation of the canonical Wnt pathway. Another study by Cheng *et al*. demonstrated that overexpression of Dkk1 prevented HSC activation and hepatic fibrosis 6. In the current study, our results demonstrated that Dkk1 was down‐regulated in HSCs isolated from fibrotic rat livers as well as TGF‐β1‐treated HSC‐T6. Furthermore, knockdown of Dkk1 by siRNA could increase the expression of β‐catenin and promote HSC activation. These findings indicate that Dkk1 acts as a potential transcriptional repressor during HSC transdifferentiation; however, the possible mechanisms by which inhibition of Dkk1 deactivates HSCs are not well understood.

Emerging evidence has shown epigenetic procedures, including DNA methylation, microRNA and histone modification, play essential roles in HSC activation 21, 22, 23, 24, 25. It has been identified that EZH2 is a typical histone methyltransferase and trimethylates H3K27me3, which results in transcriptional silencing of target genes 26. In various cancers, such as colorectal, prostate, gastric and liver cancers, EZH2 is up‐regulated 27, 28. Furthermore, EZH2‐mediated trimethylation of H3K27 is independent of DNA methylation, which leads to the transcriptional silencing of tumour suppressor genes 29. EZH2 suppresses Dkk1 transcription *via* trimethylation of H3K27me3, which has been detected in lung cancer 10. Thus, EZH2‐mediated histone methylation might play a vital role in Dkk1 transcriptional regulation during the activation of HSC. In this study, we found the levels of EZH2 were increased in both primary HSCs isolated from the liver of CCl_4_‐treated rats and TGF‐β1‐treated HSCs. Inhibition of EZH2 by DZNep reduced cell viabilities and prevented HSC activation. Additionally, knockdown of EZH2 with siRNA also prevented HSC activation. We also provided evidence that DZNep inhibited the methylation of H3K27me3 and restored the expression of Dkk1. Similar findings were found in EZH2‐knockdown HSCs. In addition, administration of DZNep inhibited the expression of β‐catenin. Consistently, knockdown of EZH2 also inhibited the expression of β‐catenin in TGF‐β1‐activated HSC‐T6. These findings suggest that EZH2 represses Dkk1 to activate Wnt/β‐catenin pathway in HSC activation.

To further confirm the essential role of Dkk1 in EZH2‐mediated activation of Wnt/β‐catenin pathway, we introduced siR‐Dkk1 with or without DZNep in activated HSC‐T6 cells, and detected the alteration of Dkk1, α‐SMA, β‐catenin and downstream target genes. Results showed that there was no difference in the expression of those genes, indicating that inhibition of EZH2 has almost no effect on the activation of HSCs when Dkk1 lacked. These results suggest that Dkk1 may be an indispensable gene in EZH2‐mediated activation of Wnt/β‐catenin pathway.

In conclusion, it was the first report regarding the epigenetic silencing of Dkk1 by histone methyltransferase EZH2 as an important mechanism mediating HSC activation and fibrogenesis. In addition, epigenetic repression of Dkk1 contributed to the up‐regulation of β‐catenin and downstream target genes, leading to HSC activation both *in vivo* and *in vitro*. This report explicitly provides evidence to better understand the functional roles of EZH2 and Dkk1 in hepatic fibrosis, which might guide the way to explore the potential targets.

## Conflict of interest

The authors confirm that there are no conflict of interest.

## Supporting information

Figure S1. H3K27me3 ChIP assay.(A) Scramble‐RNAi and si‐EZH2 were transiently transfected into treated withoutor with 10ng/ml TGF‐β in HSC‐T6 cells for 24h before lysis for aChIP procedure with normal IgG or anti‐DKK1 antibody, respectively. The H3K27me2/3 level was detected by PCR. The results are shown as relative expression against control expression without treatment. Data shown are the mean ± SD from 3 independent experiments. **P<0.01 vs control group, ##*P*<0.01 vs TGF‐β‐induced group. (B) The mRNA levels of axin‐2 in HSCs isolated from the liver of fibrosis rats and TGF‐β1‐activated HSC‐T6 cells. Data shown are the mean ± SD from 3 independent experiments. **P<0.01 vs vehicle group, ##*P*<0.01 vs control group.
