# Supplementary figures and images for: EZH2‐mediated repression of Dkk1 promotes hepatic stellate cell activation and hepatic fibrosis
Source: J Cell Mol Med. 2017 Mar 23;21(10):2317–28. doi: 10.1111/jcmm.13153 (PMC5618695; doi:10.1111/jcmm.13153)

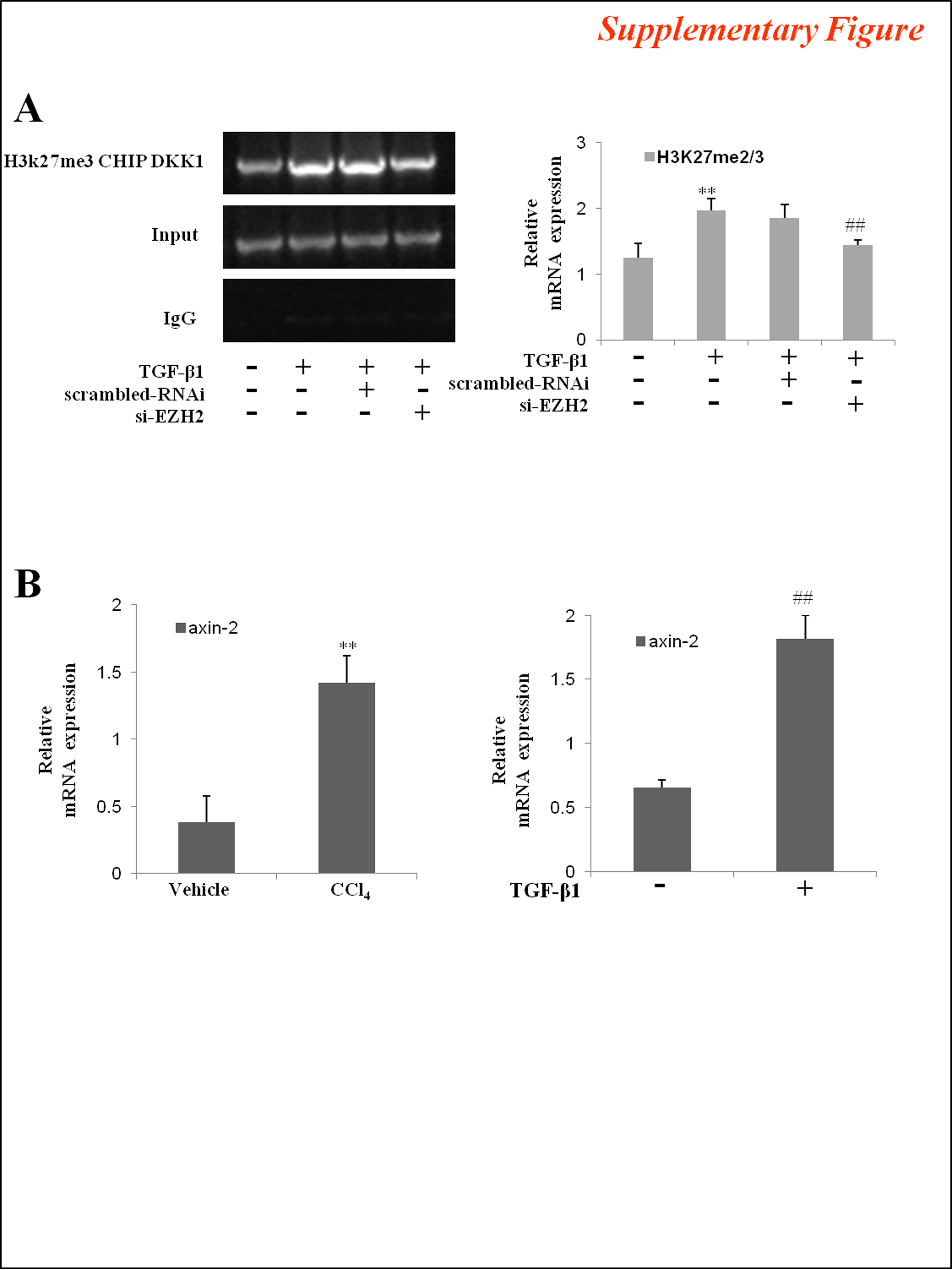

Supplement: Supplementary file 1 — Figure S1. H3K27me3 ChIP assay.(A) Scramble‐RNAi and si‐EZH2 were transiently transfected into treated withoutor with 10ng/ml TGF‐β in HSC‐T6 cells for 24h before lysis for aChIP procedure with normal IgG or anti‐DKK1 antibody, respectively. The H3K27me2/3 level was detected by PCR. The results are shown as relative expression against control expression without treatment. Data shown are the mean ± SD from 3 independent experiments. **P<0.01 vs control group, ##P<0.01 vs TGF‐β‐induced group. (B) The mRNA levels of axin‐2 in HSCs isolated from the liver of fibrosis rats and TGF‐β1‐activated HSC‐T6 cells. Data shown are the mean ± SD from 3 independent experiments. **P<0.01 vs vehicle group, ##P<0.01 vs control group. [file JCMM-21-2317-s001.tif]
